# Health-related quality of life amongst people diagnosed with abdominal aortic aneurysm and peripheral artery disease and the effect of fenofibrate

**DOI:** 10.1038/s41598-020-71454-4

**Published:** 2020-09-03

**Authors:** Jonathan Golledge, Jenna Pinchbeck, Sophie E. Rowbotham, Lisan Yip, Jason S. Jenkins, Frank Quigley, Joseph V. Moxon

**Affiliations:** 1 Queensland Research Centre for Peripheral Vascular Disease, College of Medicine and Dentistry, Australian Institute of Tropical Medicine, James Cook University, Townsville, QLD 4811 Australia; 2 The Department of Vascular and Endovascular Surgery, Townsville University Hospital, Townsville, QLD Australia; 3 Department of Vascular Surgery, The Royal Brisbane and Women’s Hospital, Brisbane, QLD Australia; 4 Mater Hospital, Townsville, QLD Australia

**Keywords:** Cardiology, Quality of life

## Abstract

The aims of this study were, firstly, to assess the effect of concurrent peripheral artery disease (PAD) on the health-related quality of life (QOL) of people diagnosed with a small abdominal aortic aneurysm (AAA); and secondly, to test whether the peroxisome proliferator-activated receptor α agonist fenofibrate improved QOL of people diagnosed with a small AAA, including those diagnosed with concurrent PAD. The study included both a cross-sectional observational study and a randomized placebo-controlled clinical trial. 140 people diagnosed with a 35–49 mm diameter AAA, 56 (40%) of whom had concurrent PAD, and 25 healthy controls were prospectively recruited. QOL was assessed with the short form (SF) 36. Findings in participants that were diagnosed with both AAA and PAD were compared separately with those of participants that had a diagnosis of AAA alone or who had neither AAA nor PAD diagnosed (healthy controls). All participants diagnosed with an AAA were then randomly allocated to 145 mg of fenofibrate per day or identical placebo. Outcomes were assessed by changes in the domains of the SF-36 and ankle brachial pressure Index (ABPI) from randomization to 24 weeks. Data were analyzed using Mann–Whitney *U* tests. Participants diagnosed with both AAA and PAD had significantly worse QOL than participants diagnosed with AAA alone or healthy controls. Fenofibrate did not significantly alter SF-36 scores or ABPI over 24 weeks. Fenofibrate does not improve QOL of people diagnosed with small AAA, irrespective of whether they have concurrent PAD.

**Trial registration**: ACTN12613001039774 Australian New Zealand Clinical Trials Registry.

## Introduction

Diseases of the abdominal aorta and its lower limb branches are common causes of leg pain, physical performance impairment and sudden death^1,2^. Two common diagnoses are abdominal aortic aneurysm (AAA) and lower limb athero-thrombotic occlusive disease (peripheral artery disease; PAD)^2,3^. People commonly have both diagnoses, with approximately 40% of people with screen-detected AAA also having a diagnosis of PAD^4,5^. Both PAD and AAA have been associated with impaired health-related quality of life (QOL)^6–8^. Whether people diagnosed with both AAA and PAD have worse QOL than those diagnosed with AAA alone is not known. It is important to clarify this, since if having diagnoses of both AAA and PAD is associated with worse QOL, this may support the value of adding screening for PAD to current AAA screening programs.

There are few medications that are effective in improving QOL of people diagnosed with either AAA or PAD. No drug has been demonstrated to improve QOL of people diagnosed with AAA^9^. Only one current medication, cilostazol, is recommended by the American^10^, but not the European^11^, guidelines to treat the leg symptoms of PAD. Cilostazol has been reported to improve some but not all the domains of the short form (SF)-36 QOL questionnaire in people with intermittent claudication^12,13^. Effective medical treatments are needed to improve QOL in people diagnosed with PAD and AAA^9,14^.

People diagnosed with PAD^15^ or AAA^16^ have microvascular dysfunction and this may contribute to impaired physical function and poor QOL. The degree of microvascular dysfunction is strongly correlated with the severity of PAD^15^. Cilostazol is thought to work by increasing nitric oxide release and improving microvascular function, further supporting the importance of microvascular dysfunction in PAD^17^. Fenofibrate is a peroxisome proliferator-activated receptor α (PPARα) agonist used to treat hypertriglyceridemia^18^. It has numerous pleiotropic effects, including upregulating nitric oxide release^19^, which might improve QOL in people diagnosed with PAD or AAA. Fenofibrate has been reported to improve angiogenesis in animal models and reduce amputations in people with diabetes in a large clinical trial^20,21^. It was therefore hypothesized that fenofibrate treatment would improve QOL in people that had been diagnosed with PAD and/ or AAA.

The current study had two aims. Aim one was to assess whether QOL was reduced in people diagnosed with both AAA and PAD in comparison to people that had between diagnosed with AAA alone or healthy controls. Aim two examined whether fenofibrate treatment for 24 weeks improved QOL of people diagnosed with AAA alone or both AAA and PAD.

## Methods

### Study design

This study included both a case–control study and a secondary analysis of a randomized control trial. In the case–control study the QOL of participants diagnosed with both AAA and PAD was compared to the QOL of participants diagnosed with AAA alone or the QOL of participants that had both an AAA and PAD excluded (healthy controls). In the second part of the study the effect of fenofibrate on the QOL of participants diagnosed with AAA was examined. The effect of fenofibrate was examined in all the participants diagnosed with AAA, whether or not they had concurrent PAD, but not the healthy controls who were excluded. This was a secondary analysis of the Fenofibrate in the Management of Abdominal Aortic Aneurysm (FAME) 2 trial. The trial protocol and primary outcome analysis of the trial have been previously published^22,23^. FAME-2 was a multi-center, parallel, double-blind clinical trial in which people with a 35–49 mm AAA were randomly allocated to receive 145 mg fenofibrate or identical placebo for 24 weeks. People were excluded if AAA repair was already planned, they were already taking fenofibrate or they had a contra-indication to fenofibrate, including liver or renal impairment, previous reaction to fenofibrate or symptomatic gallbladder disease^22^. This study was carried out according to protocols approved by the Prince Charles Hospital Human Research and Ethics Committee, the Royal Brisbane and Women’s Hospital Ethics Committee and the governance office of The Townsville Hospital and Health Services. The trial was registered with the Australian and New Zealand Clinical Trial Registry (ANZCTR12613001039774) prior to commencement. All participants provided written informed consent.

### Participants

Participants diagnosed with an AAA were recruited between the 12th October 2013 and the 11th September 2015 from vascular surgery out-patient clinics in Brisbane, Gosford and Townsville, Australia. Twenty five healthy older control participants were recruited between 26th August 2016 and 23th May 2017. Healthy controls were aged ≥ 60 years, had no history of cardiovascular disease, including ischemic heart disease, stroke and PAD, or venous disease, and an ankle brachial pressure index (ABPI) of ≥ 0.90 but < 1.4 and no diagnosis of an AAA after an abdominal ultrasound scan (infra-renal aortic diameter < 30 mm).

### Definitions, risk factors and medications

PAD was defined by a documented history of prior peripheral revascularisation for chronic limb ischemia and/ or an ABPI < 0.90. Smoking history was classified as current, former or never smoker^24^. Hypertension, diabetes, ischemic heart disease, stroke and chronic airways disease were defined by a past documented history of diagnosis or treatment for these conditions^24^. All prescribed medications were recorded at study entry. Heart rate and blood pressure was measured using a digital monitor, Omron Intellisense (HEM-907) after participants had rested for 15 min supine^25^. Recordings were measured three times and averaged. Body mass index (BMI) and waist-hip ratio were measured as previously described^24^. ABPI was measured in each lower limb using previously described methods and reported in each leg as the maximum of dorsalis pedis or posterior tibial divided by the maximum brachial pressure on either side^26^. The infra-renal aorta was imaged by ultrasound and maximum diameter measured in the anterior–posterior orthogonal plane from the outer adventitia to the outer adventitia by a single observer as previously described^23^.

### Health-related QOL

The main outcome for this study was QOL assessed with the SF-36 questionnaire which was self-administered by each participant. Item responses for the SF-36 were recoded and summed for the health domain scores of physical functioning (PF), role-physical (RP), bodily pain (BP), general health (GH), vitality (VT), social functioning (SF), role-emotional (RE) and mental health (MH)^27^. Scores were then transformed using Australian SF-36 population norms to calculate norm-based (i.e. mean of 50, standard deviation of 10) scores with 0 (worst) and 100 (best) being the lowest and highest possible scores^27^. Summary measures of physical (PCS) and mental components (MCS) were also calculated based on the 8 health domain scores^27^.

### Sample size

The sample size for FAME-2 was planned to examine the effect of fenofibrate on two circulating biomarkers at 90% power, alpha 0.025 and allowing for a drop-out rate of 20%, as previously reported^23^. It was estimated that 120 AAA participants were required. The sample size was expanded to 140 in order to minimize the effect of any incomplete adherence to study drug. No a priori sample size calculation was performed to test the effect of fenofibrate on health-related QOL as a secondary outcome of FAME-2^22^. Prior to analysis of the QOL data, however, a sample size estimate was performed. Minimal clinically important differences in SF-36 domains for people with AAA or PAD have not been defined. For another chronic health problem (back pain) a minimum clinically important difference has been estimated as 10.2% in the PCS of the SF-36^28^. In a prior study, mean (± standard deviation) results for the PCS were 36.9 ± 9.3 in 28 people with intermittent claudication^27^. Using this mean and standard deviation for the control groups of the current study, a sample size of 18 per group would have a 90% power to detect a 10.2% difference in the PCS (alpha 0.05). Based on these estimates the current study was adequately powered to test both aims.

### Data analysis

Nominal data were presented as number and percentage and were compared between groups using chi-squared test. Continuous data were not normally distributed according to the Shapiro–Wilk test and were presented as median and inter-quartile range and compared between groups using the Mann Whitney *U* test. To examine aim one, QOL domains measured with the SF-36 of participants diagnosed with both an AAA and PAD were separately compared with those of participants diagnosed with an AAA alone or healthy controls, using Mann Whitney *U* tests. To examine aim 2, changes in QOL experienced by participants enrolled in the FAME2 trial were first assessed by comparing scores for the SF-36 domains at baseline to those at 24 weeks using the related samples Wilcoxon Signed Rank Test. The change in SF-36 QOL domains over 24 weeks were then compared between participants allocated to fenofibrate and placebo using Mann Whitney U tests. In a sub-analysis, the change in SF-36 QOL domains over 24 weeks in participants that had been diagnosed with both an AAA and PAD were compared between those allocated to fenofibrate and placebo using the Mann Whitney *U* test. All analyses for aim 2 were based on intention to treat principles using all available data.

## Results

### Comparison of participants that were diagnosed with both AAA and PAD and participants diagnosed with AAA alone or healthy controls

Of the 140 participants diagnosed with AAA recruited, 56 (40%) also had PAD diagnosed. Of these 56 participants, 26 had PAD diagnosed based both on ABPI ≤ 0.90 and a prior history of PAD, 17 based on a prior history of PAD but a normal ABPI (0.91–1.20) and 13 based on ABPI ≤ 0.90 alone. The 25 healthy controls had no prior history of cardiovascular disease, a normal ABPI and no AAA. Table 1 illustrates the risk factors of participants in relation to whether they had AAA or PAD diagnosed. Most risk factors were similar for participants diagnosed with both AAA and PAD in comparison to those diagnosed with AAA alone, except that people diagnosed with both AAA and PAD were significantly more likely to have a prior history of stroke and had a significantly lower ABPI (Table 1). In comparison with participants diagnosed with both AAA and PAD, or AAA alone, the healthy controls were significantly less likely to have a number of risk factors, such as a history of smoking, hypertension, diabetes, ischemic heart disease and stroke, and were significantly less commonly prescribed medications to treat hypertension, dyslipidemia and heart disease (Table 1). Table 2 illustrates the median and inter-quartile range for the domains of the SF-36 QOL questionnaires completed at entry. Participants diagnosed with both AAA and PAD had significantly poorer QOL scores for five of the ten domains of SF-36 by comparison to participants diagnosed with AAA alone (Table 2). Participants diagnosed with both AAA and PAD had significantly poorer QOL scores for seven of the ten domains of the SF-36 by comparison with the healthy controls (Table 2).

**Table 1:** Baseline characteristics of participants in relation to diagnosis of abdominal aortic aneurysm and peripheral artery disease.

Characteristic	AAA and PAD (n = 56)	AAA no PAD (n = 84)	*P* value*	No AAA or PAD (n = 25)	*P* value†
Age (years)	78 (72–81)	75 (70–80)‡	0.232	70 (66–74)	< 0.001
Men	46 (82.1%)	71 (84.5%)	0.710	17 (68.0%)	0.157
Smoking history			0.219		< 0.001
Never	4 (7.1%)	14 (16.7%)‡		14 (56.0%)	
Former	41 (73.2%)	58 (69.0%)‡		11 (44.0%)	
Current	11 (19.6%)	12 (14.3%)‡		0 (0.0%)	
Years smoked	42 (26–54)	34 (20–49)‡	0.019	0 (0–18)	< 0.001
Average number of cigarettes per day	20 (10–25)	20 (10–25)‡	0.794	0 (0–20)	< 0.001
Hypertension	53 (94.6%)	77 (91.7%)‡	0.503	12 (48.0%)	< 0.001
Diabetes	20 (35.7%)	21 (25.0%)‡	0.172	1 (4.0%)	0.003
Ischemic heart disease	29 (51.8%)	34 (40.5%)‡	0.188	0 (0.0%)	< 0.001
Previous stroke	12 (21.4%)	8 (9.5%)	0.049	0 (0.0%)	0.012
Chronic airways disease	19 (33.9%)	20 (23.8%)	0.191	5 (20.0%)	0.205
Medications					
Anti-coagulant	8 (14.3%)	11 (13.1%)	0.840	0 (0.0%)	0.047
Calcium channel blocker	23 (41.1%)	25 (29.8%)	0.167	7 (28.0%)	0.260
Furosemide	4 (7.1%)	2 (2.4%)	0.173	0 (0.0%)	0.171
Other diuretic	12 (21.4%)	17 (20.2%)	0.865	2 (8.0%)	0.140
Beta-blocker	22 (39.3%)	34 (40.5%)	0.888	5 (20.0%)	0.089
ACE inhibitors	34 (60.7%)	39 (46.4%)	0.097	7 (28.0%)	0.007
Angiotensin receptor blockers	20 (35.7%)	32 (38.1%)‡	0.775	3 (12.0%)	0.029
Any anti-platelet	41 (73.2%)	54 (64.3%)‡	0.268	5 (20.0%)	< 0.001
Statin	47 (83.9%)	65 (77.4%)‡	0.343	7 (28.0%)	< 0.001
Body mass index (Kg/m^2^)	27.00 (23.00–31.00)§	27.50 (24.00–32.00)	0.389	25.66 (23.75–29.54)	0.600
Waist-hip ratio	1.00 (0.95–1.04)§	0.98 (0.92–1.02)	0.232	0.95 (0.89–1.03)	0.049
Left ABPI	0.90 (0.71–1.04)	1.07 (1.00–1.14)‡	< 0.001	1.15 (1.06–1.20)	< 0.001
Right ABPI	0.86 (0.65–1.00)	1.06 (1.00–1.16)‡	< 0.001	1.14 (1.08–1.19)	< 0.001
Systolic blood pressure (mmHg)	144 (133–153)	140 (127–152)‡	0.109	123 (118–133)	< 0.001
Diastolic blood pressure (mmHg)	75 (70–83)	77 (70–82)	0.774	76 (70–79)	0.806
Heart rate (beats per minute)	64 (58–71)	67 (60–72)	0.398	61 (59–70)	0.842
Infra-renal aortic diameter (mm)	39.9 (37.3–43.7)	39.2 (35.9–43.2)	0.433	18.2 (16.3–21.8)**‖**	< 0.001

*AAA* abdominal aortic aneurysm, *PAD* peripheral artery disease, *ABPI* ankle-brachial pressure index.

*Comparisons of participants who had both AAA and PAD diagnosed with those who had AAA but not PAD diagnosed were performed with chi-squared and Mann–Whitney *U* tests. †Comparison of participants who had both AAA and PAD diagnosed with those who had neither diagnosed were performed with chi-squared and Mann–Whitney *U* tests. ‡*P* < 0.05 for comparisons between participants with AAA but not PAD diagnosed and those with neither AAA nor PAD diagnosed.

Missing from 1§ and 5**‖** participants.

**Table 2:** Comparison of the health-related quality of life of participants in relation to the diagnosis of abdominal aortic aneurysm and lower limb peripheral artery disease.

Short-form 36 domain	AAA and PAD	AAA no PAD	*P* value*	No AAA or PAD	*P* value†
Physical functioning	37 (32–48)	49 (38–53)	< 0.001	51 (46–53)	< 0.001
Role physical	48 (41–54)	52 (42–56)	0.024	54 (50–56)	0.011
Bodily pain	44 (36–59)	48 (40–59)	0.354	53 (46–59)	0.241
General health	44 (32–51)	50 (39–55)	0.003	50 (46–56)	0.001
Vitality	47 (37–55)	52 (43–55)‡	0.039	55 (49–58)	0.001
Social functioning	57 (45–57)	57 (51–57)‡	0.154	57 (57–57)	0.016
Role emotional	55 (47–55)	55 (47–55)‡	0.328	55 (51–55)	< 0.001
Mental health	55 (46–58)	58 (49–60)	0.101	58 (49–61)	0.276
Physical component summary	41 (32–48)	48 (37–53)‡	0.002	51 (48–55)	< 0.001
Mental component summary	56 (50–60)	57 (51–61)	0.572	58 (53–61)	0.363

Shown are median (inter-quartile range) scores for the different domains of the Short-Form 36 questionnaire. *Comparisons of participants who had both AAA and PAD diagnosed with those who had AAA but not PAD diagnosed were performed with chi-squared and Mann–Whitney *U* tests. †Comparison of participants who had both AAA and PAD diagnosed with those who had neither diagnosed were performed with chi-squared and Mann–Whitney *U* tests. ‡*P* < 0.05 for comparisons between participants with AAA but not PAD diagnosed and those with neither AAA nor PAD diagnosed.

*AAA* abdominal aortic aneurysm, *PAD* peripheral artery disease.

### Effect of fenofibrate on QOL and ABPI

**Figure 1:**
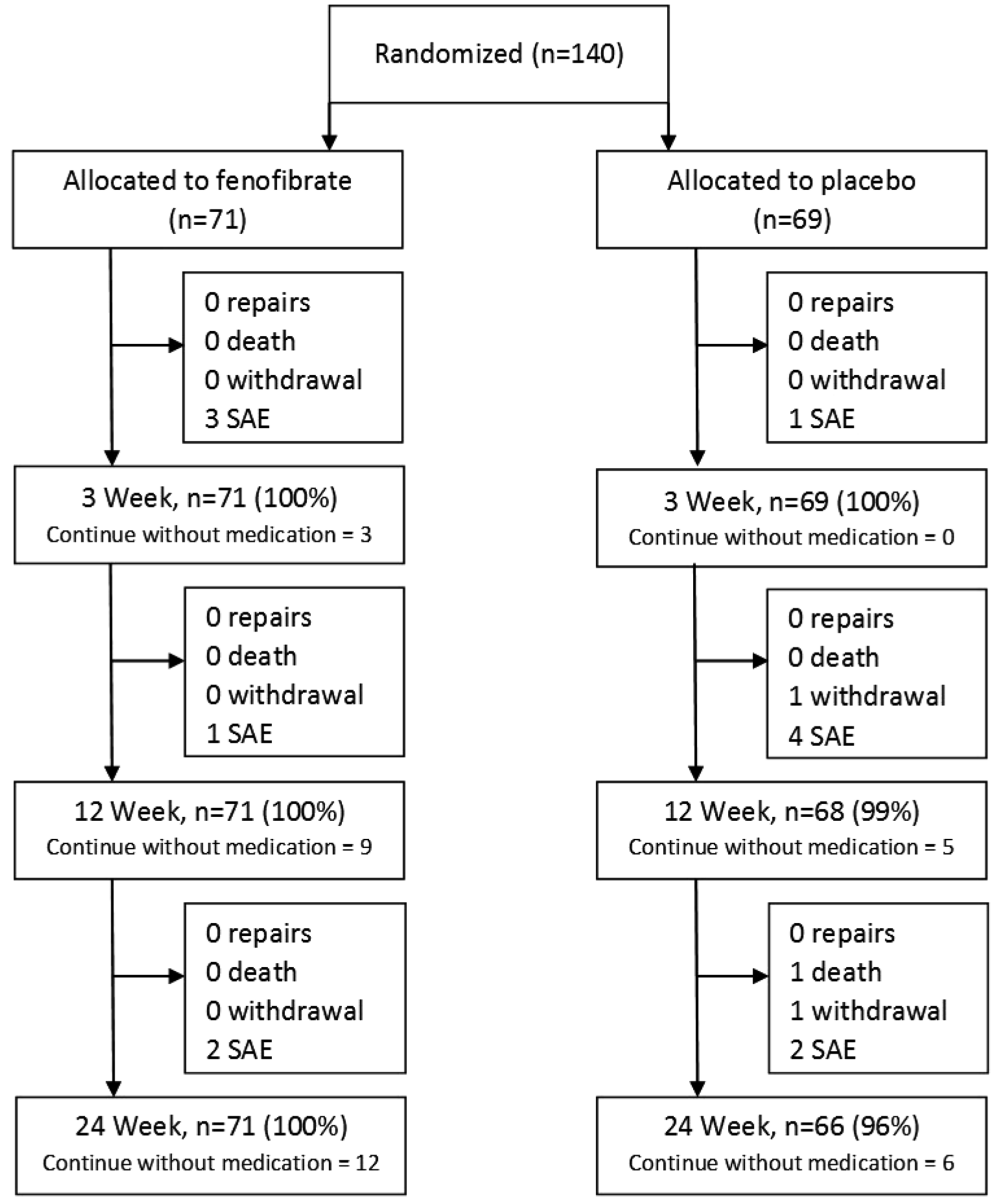
Illustration of the participant flow in the FAME-2 trial as previously published^23^.

The 140 participants diagnosed with AAA, not the healthy controls, were randomized to fenofibrate or placebo. One hundred and thirty seven of these 140 (98%) participants completed the 24 week FAME-2 study. There was one death and two withdrawals amongst the participants allocated placebo (See Fig. 1)^23^. Pill counting suggested that 119 (85%) of participants took ≥ 80% of their medications, with no significant difference between groups^23^. As previously reported, participants allocated fenofibrate had significantly lower serum triglyceride after 3 and 24 weeks in comparison to those allocated placebo and no excess of adverse events^23^.

Over the 24 weeks of the study the participants had a small but significant decrease in median scores for two domains of the SF-36—the vitality (VT) and mental health (MH) domains (Table 3). No significant differences in the magnitude of change from baseline scores in any of the QOL domains were observed when comparing participants allocated fenofibrate or placebo (Table 4). These findings were similar whether all participants were analysed or just those diagnosed with both AAA and PAD (Table 4). Fenofibrate also had no effect on change in ABPI over 24 weeks (Table 4).

**Table 3:** Comparison of the health-related quality of life of participants enrolled in the FAME-2 trial at baseline and 24 weeks.

Short-form 36 domain	Baseline	24 weeks	*P* value*
Physical functioning	44 (33–51)	44 (31–51)	0.469
Role physical	50 (41–56)	52 (41–56)	0.371
Bodily pain	46 (36–59)	48 (36–53)	0.471
General health	48 (37–55)	48 (35–53)	0.250
Vitality	49 (43–55)	46 (40–52)	0.014
Social functioning	57 (51–57)	57 (48–57)	0.730
Role emotional	55 (47–55)	55 (47–55)	0.444
Mental health	55 (49–58)	55 (46–58)	0.025
Physical component summary	44 (35–51)	44 (35–51)	0.993
Mental component summary	57 (51–60)	56 (49–60)	0.050

Shown are median (inter-quartile range) scores for the different domains of the Short-Form 36 questionnaire.

*P values were generated using the related samples Wilcoxon Signed Rank test.

**Table 4:** Comparison of change in the health-related quality of life of participants allocated fenofibrate or placebo over 24 weeks.

All participants			
Short-form 36 domain	Drug allocation		*P* values
	Fenofibrate (n = 70)	Placebo (n = 67)	
Physical functioning	0 (− 4 to 4)	0 (− 4 to 4)	0.566
Role physical	0 (− 2 to 4)	0 (− 2 to 4)	0.714
Bodily pain	0 (− 8 to 4)	0 (− 4 to 5)	0.061
General health	0 (− 2 to 5)	0 (− 7 to 2)	0.166
Vitality	0 (− 6 to 3)	0 (− 6 to 3)	0.965
Social functioning	0 (0 to 0)	0 (0 to 0)	0.776
Role emotional	0 (− 6 to 0)	0 (− 3 to 5)	0.511
Mental health	0 (− 6 to 3)	0 (− 6 to 3)	0.718
Physical component summary	− 1 (− 4 to 3)	0 (− 4 to 4)	0.532
Mental component summary	0 (− 5 to 2)	− 1 (− 6 to 3)	0.880
**Haemodynamic outcomes**			
Percentage change in left ankle-brachial pressure index	− 0.9 (− 5.3 to 1.3)‡	− 0.7 (− 6.7 to 3.2)*	0.927
Percentage change in right ankle-brachial pressure index	− 1.0 (− 7.9 to 2.2)‡	− 0.4 (− 6.1 to 4.7)†	0.406

Participants diagnosed with both AAA and PAD			
Short-Form 36 domain	Drug allocation		*P* values
	Fenofibrate (n = 24)	Placebo (n = 29)	
Physical functioning	1 (− 2 to 4)	2 (− 1 to 6)	0.346
Role physical	0 (0 to 5)	0 (− 4 to 4)	0.349
Bodily pain	− 2 (− 8 to 3)	0 (− 4 to 6)	0.142
General health	− 1 (− 5 to 3)	0 (− 4 to 3)	0.529
Vitality	− 5 (− 9 to 3)	0 (− 6 to 5)	0.285
Social functioning	0 (− 6 to 0)	0 (− 3 to 6)	0.342
Role emotional	0 (− 4 to 0)	0 (0 to 8)	0.196
Mental health	− 1 (− 9 to 0)	0 (− 6 to 4)	0.068
Physical component summary	0 (− 3 to 4)	0 (− 5 to 4)	0.915
Mental component summary	− 1 (− 7 to 1)	1 (− 5 to 4)	0.174
**Haemodynamic outcomes**			
Percentage change in left ankle-brachial pressure index change	− 0.8 (− 3.2 to 0.6)§	0.2 (− 5.7 to 3.5)**‖**	0.612
Percentage change in right ankle-brachial pressure index change	0.1 (− 6.2 to 11.8)§	− 1.6 (− 6.6 to 1.9)**‖**	0.352

Shown are median (inter-quartile range) changes in scores for different domains of the Short-Form 36 questionnaire over 24 weeks. Three participants diagnosed with both AAA and PAD allocated to placebo were lost to follow-up and did not complete SF-36 assessments at 24 weeks. In some participants ABPI could not be measured due to incompressible arteries and thus assessment were limited to †65, *66, ‡68, §23 or **‖**29 participants.

## Discussion

This study demonstrates that people diagnosed with both AAA and PAD have lower scores for several domains of the SF-36 by comparison to those diagnosed with AAA but not PAD. The study also found that, within a placebo-controlled randomized controlled trial, fenofibrate treatment for 24 weeks did not improve QOL or ABPI amongst participants diagnosed with AAA, whether or not they had concurrent PAD diagnosed.

International guidelines and systematic reviews recommend that patient-reported outcome measures receive greater attention^11,29^. This study confirms that people diagnosed with AAA have worse QOL than healthy controls^8^. Similar to previous studies, 40% of the participants diagnosed with AAA had concurrent PAD diagnosed^4,5^. QOL was worse in participants that had diagnoses of both PAD and AAA than in those that had a diagnosis of AAA alone. One possible explanation for this could be the greater co-morbidities in participants with both vascular problems. Prior history of stroke, but not other co-morbidities, such as chronic airways disease and ischemic heart disease, was more common in participants that had diagnoses of both lower PAD and AAA by comparison to those that just had a diagnosis of AAA. It is possible that this difference contributed to the poorer physical aspects of QOL in participants diagnosed with both vascular conditions. More likely, though, is that the established effect of leg ischemia to cause pain and reduce physical performance was responsible for the differences in QOL demonstrated^7,8^. People diagnosed with both AAA and PAD are also substantially more likely to have a cardiovascular event, with a recent study reporting that those with concurrent PAD had about a threefold higher rate of major cardiovascular events than those diagnosed with AAA alone^30^. Given the high prevalence, worse QOL and increased cardiovascular event rate associated with concurrent PAD amongst people diagnosed with AAA, there is a good case for screening for low ABPI or absent pulses amongst people identified to have AAA, although this is not currently recommended in European guidelines^11,31^. Diagnosis of PAD would enable additional treatment, such as supervised exercise therapy, to be provided aimed at improving QOL.

Prior animal and clinical observational studies suggest that fenofibrate has potential to improve QOL amongst people diagnosed with PAD and/or AAA^15–17,19–21^. This study, however, found no evidence of benefit of fenofibrate on aspects of QOL assessed by the SF-36 in participants diagnosed with AAA alone or both AAA and PAD. Fenofibrate also had no effect on ABPI over 24 weeks. These findings do not favour further investigation of fenofibrate as a medication to improve QOL in people diagnosed with AAA.

The findings of this study should be interpreted after considering the strengths and weaknesses of the investigation. This was the first study, as far as the investigators are aware, to compare QOL in people diagnosed with both AAA and PAD to those diagnosed with AAA alone. The sample sizes included were small and the generalizability of the findings needs to be more widely examined. Also assessment of QOL was limited to the generic SF-36. While this is a commonly used measure, a number of new patient reported outcome assessments have now been developed for people diagnosed with AAA but these were not available for the current study^32^. In testing the effect of fenofibrate on QOL an important strength was the randomized placebo-controlled design. Blinding of participants and investigators was maintained throughout. Adherence to study medication was good and loss to follow-up low. Important weaknesses included that QOL was a secondary outcome of FAME-2 and thus no a priori sample size estimate was performed. While the posthoc sample size estimate suggested the study was adequately powered, it remains possible, particular in the sub-analysis of people diagnosed with both AAA and PAD, that the study was underpowered. Also no objective assessment of physical performance was performed. This investigation therefore does not rule out a moderate effect of fenofibrate in improving (or reducing) QOL in people with PAD and/or AAA. It should also be acknowledged that this was a secondary analysis of a trial for which the primary outcome analysis was negative and therefore the findings need to be interpreted as exploratory.

In conclusion, this study suggests that amongst people diagnosed with a small AAA, those who also had a concurrent diagnosis of PAD had the worst health-related QOL. The study also found no benefit of fenofibrate in improving QOL in people diagnosed with a small AAA, irrespective of whether they had concurrent PAD diagnosed.
